# LRRC25 Functions as an Inhibitor of NF-κB Signaling Pathway by Promoting p65/RelA for Autophagic Degradation

**DOI:** 10.1038/s41598-017-12573-3

**Published:** 2017-10-18

**Authors:** Yanchun Feng, Tianhao Duan, Yang Du, Shouheng Jin, Mingjun Wang, Jun Cui, Rong-Fu Wang

**Affiliations:** 1 Zhongshan School of Medicine, Sun Yat-sen University, Guangzhou, 510080 China; 2 Key Laboratory of Gene Engineering of the Ministry of Education, State Key Laboratory of Biocontrol, School of Life Sciences, Sun Yat-sen University, Guangzhou, 510275 China; 3 Shenzhen Institute for Innovation and Translational Medicine, Shenzhen, 518120 China; 4 Center for Inflammation and Epigenetics, Houston Methodist Research Institute, Houston, Texas 77030 USA; 5 Department of Microbiology and Immunology, Weill Cornell Medicine, Cornell University, New York, NY 10065 USA

## Abstract

Nuclear factor κB (NF-κB) is a family of critical transcription factors that play a critical role in innate immune responses and inflammation, yet the molecular mechanisms responsible for its tight regulation is not fully understood. In this study, we identified LRRC25, a member of leucine-rich repeat (LRR)-containing protein family, as a negative regulator in the NF-κB signaling pathway. Ectopic expression of LRRC25 impaired NF-κB activation, whereas knockout of *LRRC25* potentiated NF-κB activation and enhanced the production of inflammatory cytokines. Further study demonstrated that the LRR domain of LRRC25 interacted with the Rel Homology domain (RHD) of p65/RelA and promotes the degradation of p65/RelA. Furthermore, LRRC25 enhanced the interaction between p65/RelA and cargo receptor p62, thus facilitating the degradation of p65/RelA through autophagy pathway. Our study has not only identified LRRC25 as a novel inhibitor of NF-κB signaling pathway, but also uncovers a new mechanism of crosstalk between NF-κB signaling and autophagy pathways.

## Introduction

The innate immune system is orchestrated by several pattern recognition receptors (PRRs), including Toll-like receptors (TLRs), nucleotide-binding domain (NOD)-like receptors (NLRs), and retinoic acid-inducible gene I (RIG-I)-like receptors (RLRs)^1,2^. Detection of pathogen-associated molecular patterns (PAMPs) of invading pathogens by PRRs results in the activation of downstream pathways to induce the expression of pro-inflammatory and type I interferons (IFNs) genes^1^. However, if excessive activation occurs, it could lead to, fatal bacterial sepsis, autoimmune and chronic inflammatory diseases. Therefore, tight negative regulation of innate immune signaling pathways is crucial towards maintaining the homeostasis of immune responses^3,4^.

The nuclear Factor -κB (NF-κB) signaling pathway can be activated by different TLR ligands, tumor necrosis factor alpha (TNF-α) and interleukin-1β (IL-1β), resulting in the recruitment of adaptor proteins such as myeloid differentiation primary response gene 88 (MyD88), receptor-interacting protein (RIP1), and TIR-domain-containing adapter-inducing interferon-β (TRIF)^5^. These proteins act on downstream tumor necrosis factor receptor (TNF-R)-associated factor (TRAF) signaling molecules including TRAF6, TRAF3, TRAF2 and TRAF5, which synthesize multiple poly-ubiquitin chains on themselves or other molecules, and recruit TGF-beta-activated kinase 1 (TAK1) and IκB kinase (IKK) complex^6,7^. The IKK complex consists of catalytic subunits IKKα and IKKβ, and the NF-κB essential modulator (NEMO), also known as IKKγ. Activated IKK complex phosphorylates IκB proteins at two N-terminal serine residues (S32 and S36), triggering their ubiquitination and proteasomal degradation. Degradation of IκB release and allow NF-κB to translocate into the nucleus, resulting in transcription of NF-κB-mediated genes^1,8,9^.

There are five members of the NF-κB transcription factors: p50, p52, p65/RelA, c-Rel, and RelB proteins^3^. All of these proteins share an N-terminal Rel homology domain (RHD) that mediates DNA binding and homo- and heterodimerization. p65/RelA, c-Rel, and RelB contain transcription activation domains (TADs). which are responsible for positively regulating the expression of downstream genes. p50 and p52, which lack TADs, mainly inhibit transcription, unless they are recruited by other coactivators or interaction with a TAD-containing NF-κB member^10^. IκB proteins consist of IκBα, IκBβ, IκBε, IκBγ, Bcl3 and IκBζ. IκBα, IκBβ, IκBε, and IκBγ function as inhibitors that associate with NF-κB dimers, while Bcl3 and IkBζ influence NF-κB transactivation in the nucleus^11–13^. Upon the activation of the IKK complex, the IκBs can be rapidly degraded to release NF-κB dimers into the nucleus, which, in turn, activates the expression of subsequent NF-κB-mediated genes^10^.

LRRs are present many prokaryotic and eukaryotic proteins and crucial to the innate immune system through mediating protein-protein interactions^14^. In terms of innate immune sensing, detection of PAMPs via PRRs characteristically involves LRRs. However, other LRR-containing receptors such as TLRs and NLRs, functions of many members of the human LRR-containing proteins in the innate immunity, are poorly defined^14,15^. In order to clearly define the roles of LRR-containing proteins in NF-κB signaling pathway, we carried out a functional screening and identified LRRC25 as a potent negative regulator of NF-κB signaling. In addition, we demonstrated that LRRC25 functions as an inhibitor of NF-κB signaling by promoting p65/RelA for autophagy degradation.

## Results

### Identification of LRRC25 as a negative regulator of NF-κB signaling

**Figure 1:**
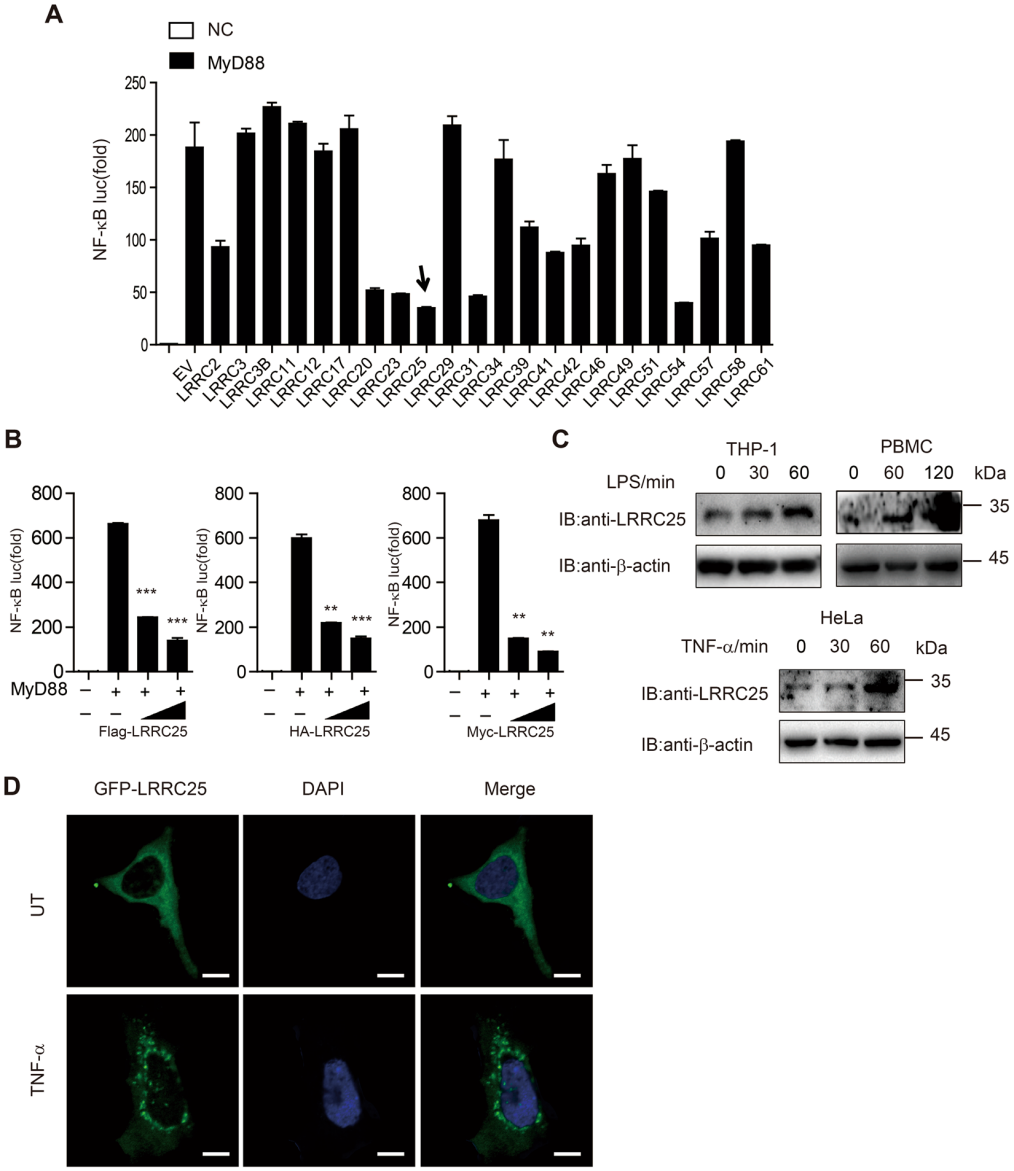
Identification of LRRC25 as a Negative Regulator of NF-κB Signaling. (**A**) HEK293T cells were transfected with plasmids of 22 LRRCs, empty vector (EV) and a NF-κB-luc reporter plasmid, together with or without (negative control, NC) MyD88 and analyzed for NF-κB activity. (**B**) HEK293T cells were transfected with plasmids of Flag-tagged, HA-tagged, and Myc-tagged-LRRC25, MyD88 and a NF-κB-luc reporter plasmid and analyzed for NF-κB activity. Values are means ± SEM (n = 3) of three independent experiments (*p < 0.05, **p < 0.01, ***p < 0.001). (**C**)THP-1 cells and PBMCs were treated with 200 ng/ml LPS, and HeLa cells were treated with 20 ng/ml TNF-α at the indicated time points. Cells lysates were subjected to immunoblotting with the indicated antibodies. Unprocessed original scans of blots are shown in Supplementary Fig. S3. Data are representative of three independent experiments. (**D**) Confocal microscopy of HeLa cells transfected with GFP-LRRC25 for 24 hrs and then treated with 20 ng/ml TNF-α for 45 min. DAPI (blue) was used for nuclear staining, Scale bar: 10 μm.

We investigated the roles of LRRC family proteins in the regulation of NF-κB signaling by co-transfecting expression vectors for individual LRRC proteins with a NF-κB luciferase reporter and Flag-tagged MyD88, which can induce activation of NF-κB-luc. Among these candidate proteins, we identified LRRC25 as a potent inhibitor of MyD88-induced NF-κB activation (Figs 1A and S1A). Similar results were obtained with LRRC25 with different tags (Fig. 1B). To determine whether the LRRC25 expression could be altered in response to NF-κB activation, we treated THP-1 cells, peripheral blood mononuclear cells (PBMCs) and HeLa cells with lipopolysaccharide (LPS) or tumor necrosis factor (TNF-α) to activate the NF-κB pathway. Immunoblot (IB) analysis revealed that LRRC25 protein level was strongly upregulated by LPS or TNF-α treatment (Fig. 1C).

We next investigated whether the increased protein abundance of LRRC25 was due to the elevated *LRRC25* mRNA level after NF-κB activation. Real-time PCR analysis showed that the mRNA level of *LRRC25* was not changed upon LPS treatment in THP-1 cells (Supplementary Fig. S1B), indicating that LRRC25 protein amount was increased at the post-translational level after LPS treatment. Further experiments revealed that ectopic expression of p65 could inhibit K48-linked ubiquitin chains of LRRC25, suggesting that NF-κB signaling may stabilize LRRC25 by inhibiting LRRC25 K48-linked ubiquitination for degradation (Supplementary Fig. S1C). To determine the cellular localization of LRRC25 upon stimulation, we transfected HeLa cells with GFP-tagged LRRC25 (GFP-LRRC25) fusion construct, and found that LRRC25 showed a punctate appearance in the cytoplasm after cells were treated with TNF-α for 45 min (Fig. 1D).

### LRRC25 inhibits NF-κB signaling pathway and inflammatory response

**Figure 2:**
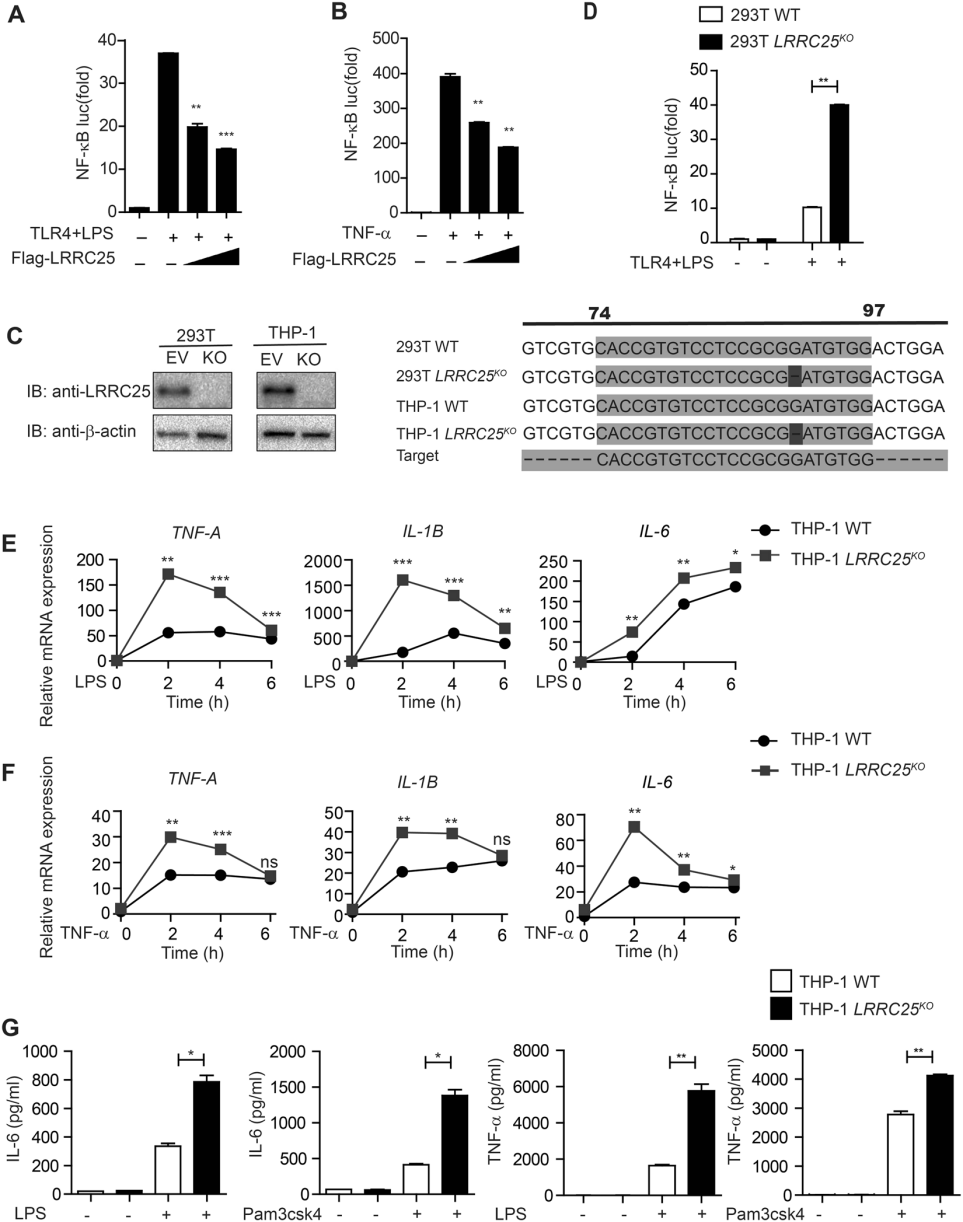
LRRC25 Inhibits NF-κB Activation and Impairs the Inflammatory Response. (**A**,**B**,**D**) HEK293T^WT^ or 293T *LRRC25* ^*KO*^ cells were transfected with a NF-κB-luc reporter plasmid, a TLR4 plasmid (for LPS treatment), and an empty vector or LRRC25 construct and analyzed for NF-κB luciferase activity after treatment with 10 μg/ml LPS for 12 h (**A**,**D**) and 20 ng/ml TNF-α for 6 h (**B**). (**C**) Protein extracts of HEK293T^WT^ and 293T *LRRC25* ^*KO*^ cells, THP-1^WT^ or THP-1 *LRRC25* ^*KO*^ cells were subjected to immunoblot with anti-LRRC25 antibody, with the sequence alignment of 293T *LRRC25* ^*KO*^ with 293T^WT^, and THP-1 *LRRC25* ^*KO*^ cells with WT. Unprocessed original scans of blots are shown in Supplementary Fig. S3. (**E**) THP-1^WT^ or THP-1 *LRRC25* ^*KO*^ cells were treated with LPS (200 ng/ml) for 0, 2, 4, 6 h. Total RNAs from the treated cells were harvested at the indicated time points and mRNA level of *TNF-*α, *IL-1*β, and *IL-6* were determined by real-time PCR analysis. (**F**) THP-1^WT^ or THP-1 *LRRC25* ^*KO*^ cells were treated with or without TNF-α (20 ng/ml) for the indicated time. The mRNA levels of *TNF-*α, *IL-1*β and *IL-6* were detected by real-time PCR analysis. (**G**) THP-1^WT^ or THP-1 *LRRC25* ^*KO*^ cells were treated with LPS or Pam3csk4 for 24 h. Cells supernatant were then collected to measure the IL-6 and TNF-α production by ELISA. Data in figure (**A**–**G**) are means ± SEM (n = 3) of three independent experiments (*p < 0.05, **p < 0.01, ***p < 0.001).

Next, we determined whether LRRC25 inhibits NF-κB activation induced by LPS or TNF-α treatment. We transfected 293T or 293T/TLR4 cells with plasmids encoding the NF-κB-luc reporter, the renilla luciferase reporter (*pRL-TK-luc*) (an internal control) and Flag-tagged LRRC25. The cells were treated with LPS or TNF-α, respectively. We found that the activity of NF-κB-luc induced by both different stimuli could be inhibited by LRRC25 (Fig. 2A,B).

To further define the function of LRRC25, we generated the *LRRC25* knockout KO) 293T cells (*LRRC25* ^*KO*^ 293T) and THP-1 cells (*LRRC25* ^*KO*^ THP-1 cells) using the CRISPR/Cas9 system (Fig. 2C). To demonstrate the effects of *LRRC25* ^KO^ cells on the activation of NF-κB pathway, we transfected the wild type 293T cells (293T^WT^) and *LRRC25* ^*KO*^ 293T cells with plasmids expressing NF-κB-luc and *pRL-TK-lu*c, and found that *LRRC25* knockout resulted in much higher activities of LPS-induced NF-κB-luc reporter (Fig. 2D).

To determine whether increased NF-κB activation in *LRRC25* KO cells could up-regulate the expression of NF-κB-responsive cytokines, we measured the mRNA levels of several pro-inflammatory cytokines in wild type THP-1 cells (THP-1WT) and *LRRC25* ^*KO*^ THP-1 cells. Upon LPS treatment, *LRRC25* ^*KO*^ THP-1 cells exhibited elevated expression of inflammatory cytokines including *TNF-*α, *IL-1*β, and *IL-6*, as compared to THP-1WT cells (Fig. 2E). Further, we found that expression level of TNF-α, IL-1β and IL-6 was markedly increased in *LRRC25* ^*KO*^ THP-1 cells compared to control cells after TNF-α treatment (Fig. 2F). IL-6 and TNF–α protein levels were also markedly increased in *LRRC25* ^*KO*^ THP-1 cells compared to control cells after LPS or Pam3csk treatment (Fig. 2G). Taken together, these results suggest that LRRC25 is a potent negative regulator of NF-κB signaling pathway.

### LRRC25 inhibits NF-κB activation at p65/RelA level

**Figure 3:**
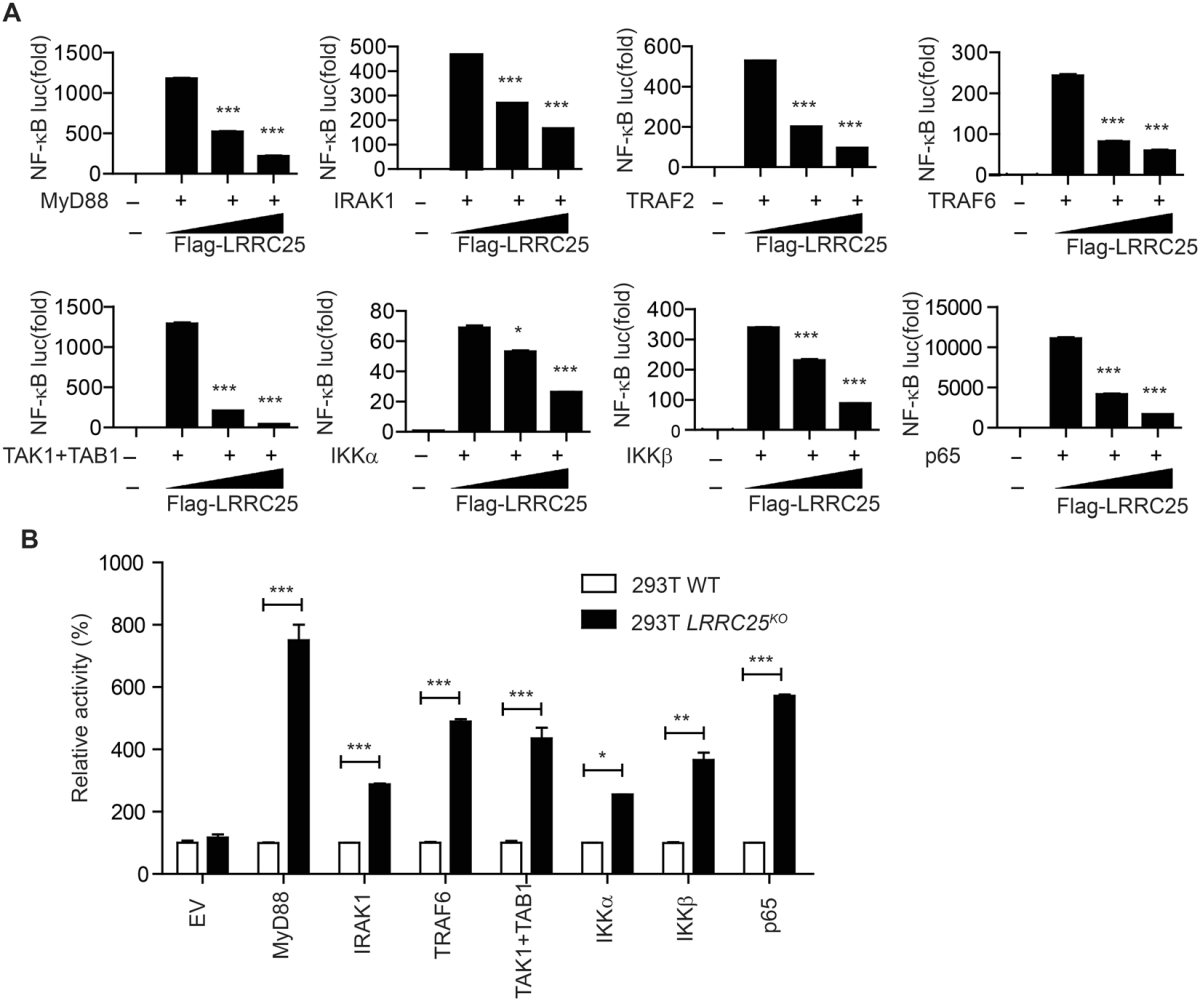
LRRC25 Inhibits NF-κB Activation at p65/RelA Level. (**A**) HEK293T cells were transfected with a NF-κB-luc reporter and plasmids encoding MyD88, IRAK1, TRAF2, TRAF6, TAK1 + TAB1, IKKα, IKKβ or p65/RelA, along with empty vector (no wedge) and increasing amount (wedge) of expression vector for LRRC25 (0, 50, 100 ng) for 24 h and analyzed for NF-κB luciferase activity. (**B**) HEK293T^WT^ and 293T *LRRC25* ^*KO*^ cells were transfected with a NF-κB-luc reporter, plus plasmids of MyD88, IRAK1, TRAF2, TRAF6, TAK1 + TAB1, IKKα, IKKβ or p65/RelA for 24 h and analyzed for NF-κB luciferase activity. Data in (A-B) are means ± SEM of three independent experiments (*p < 0.05, **p < 0.01, ***p < 0.001).

We next sought to determine the molecular mechanisms by which LRRC25 inhibits NF-κB signaling pathway. We transfected 293T cells with expression vectors encoding MyD88, IRAK1, TRAF2, TRAF6, TAK1 + TAB1, IKKα, IKKβ or p65/RelA, together with increasing amounts of *LRRC25* expression vector plus the NF-κB luciferase reporter, and then measured the effect of LRRC25 expression on NF-κB-luc activity. We found that NF-κB activation induced by MyD88, IRAK1, TRAF2, TRAF6, TAK1 + TBK1, IKKα, IKKβ or p65/RelA was strongly inhibited by LRRC25 in a dose-dependent manner (Fig. 3A). Consistently, knockout of *LRRC25* in 293T cells enhanced NF-κB-luc activities induced by MyD88, IRAK1, TRAF2, TRAF6, TAK1 + TBK1, IKKα, IKKβ, or p65/RelA (Fig. 3B). These results suggest that LRRC25 inhibits NF-κB signaling through p65/RelA.

### LRRC25 promotes the degradation of p65/RelA

**Figure 4:**
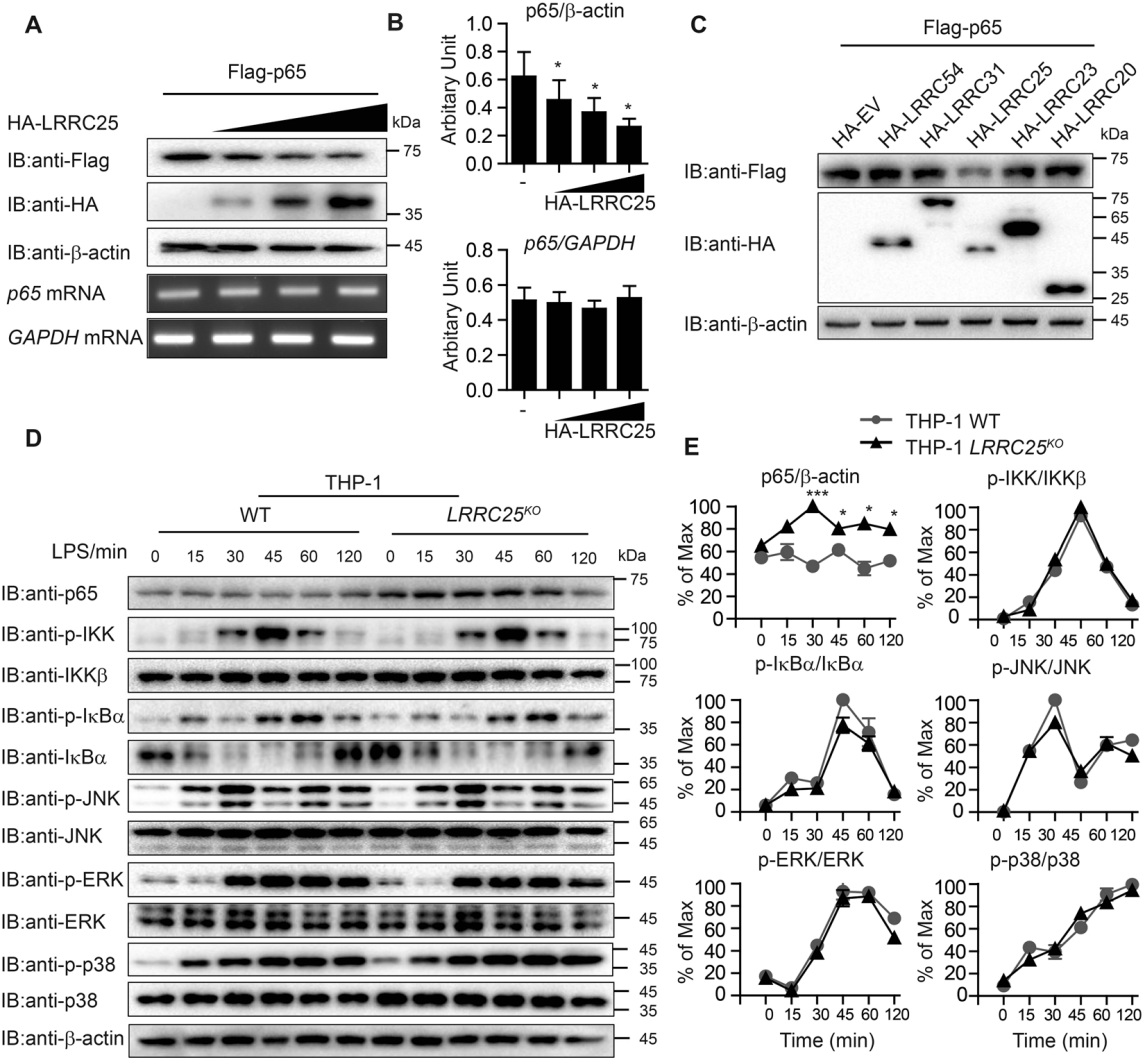
LRRC25 Mediates the Degradation of p65/RelA. (**A**) Immunoblot analysis of protein extracts of 293T cells transfected with empty vector or vector for HA-LRRC25 in an increasing amount (wedge), along with expression plasmids of Flag-p65/RelA. Below, real-time analysis of *RelA* mRNA: *GAPDH* (encoding glyceraldehyde phosphate dehydrogenase) was used as a loading control. Repeated blots are shown in Supplementary Fig. S1D. (**B**) Quantification of the protein level of p65 to β-actin is shown at the top and mRNA of *p65* to *GAPDH* at the bottom. Data are normalized to β-actin (top) or *GAPDH* (bottom) and shown as mean ± SEM (n = 3), student’s t-tests, *p < 0.05, **p < 0.01. (**C**) Immunoblot analysis of protein extracts of 293T cells transfected with empty vector or vector for Flag-p65/RelA, along with plasmids of HA-LRRC20, HA-LRRC23, HA-LRRC25, HA-LRRC31, or HA-LRRC54. Repeated blots are shown in Supplementary Fig. S2. (**D**,**E**) THP-1^WT^ and *LRRC25* ^*KO*^ THP-1 cells were treated with LPS (200 ng/ml) at the indicated time points. The expression of p65/RelA and phospho-IκBα, total IκBα, phospho-IKK, total IKK, phospho-JNK, total JNK, phospho-ERK, total ERK, phospho-p38, and total p38 were analyzed by immunoblotting with the indicated antibodies. Repeated blots are shown in Supplementary Fig. S1E. Quantitative comparison of protein expression and signaling activation between THP-1^WT^ and *LRRC25* ^*KO*^ THP-1 cells by scanning of blots in (**E**). Data are normalized to THP-1^WT^ cells and shown as mean ± SEM (n = 3), Student’s t-tests, *p < 0.05, **p < 0.01. Unprocessed original scans and of blots are shown in Supplementary Fig. S3.

We next investigated how LRRC25 could exert its negative effect at the p65/RelA level. We transfected 293T cells with plasmids encoding Flag-tagged p65/RelA and HA-tagged LRRC25, and found that the abundance of p65/RelA protein was diminished when LRRC25 was expressed (Fig. 4A, top). To exclude the possibility that the down-regulation of p65/RelA protein level was caused by a lower expression of the gene *RelA*, we performed real-time PCR analysis and found that the abundance of p65/RelA mRNAs was not changed with increasing expression of LRRC25 (Fig. 4A). Quantification of band density scanning of the blots in Fig. 4A showed that the protein level, but not the mRNA, of p65 was reduced by LRRC25 in a dose-dependent manner (Fig. 4B). In addition, we found that LRRC25, but not other LRRC proteins, such as LRRC20, LRRC23, LRRC31 and LRRC54, could specifically reduce the protein level of p65/RelA (Fig. 4C). Consistent with these observations, knockout of *LRRC25* resulted in increase of endogenous p65/RelA in THP-1 cells (Fig. 4D,E). By contrast, IB analysis showed little or no appreciable difference in the phosphorylation level and protein abundances of IκBα and MAPKs (JNK1/2, ERK1/2 and p38) between wild type and *LRRC25* ^*KO*^ THP-1 cells (Fig. 4D,E). Furthermore, we found that LRRC25 also mediated the degradation of p105/p50 but not p100/p52 (Supplementary Fig. S1F), indicating that LRRC25 inhibits NF-κB signaling by targeting p65/p50 heterodimer for the degradation.

### LRRC25 interacts with p65/RelA

**Figure 5:**
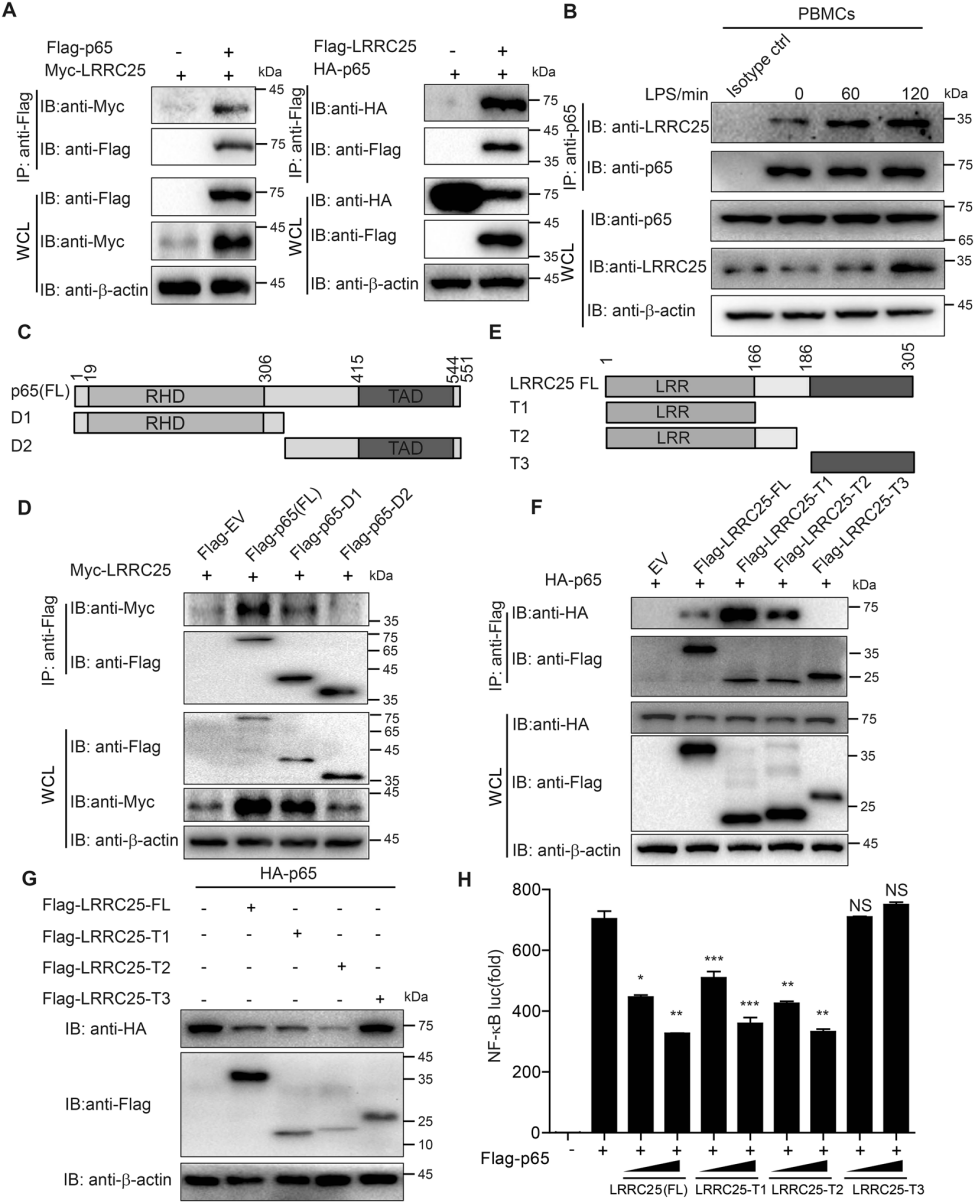
LRRC25 Interacts with p65/RelA. (**A**) Flag-p65/RelA and Myc-LRRC25 expression plasmids were transfected into HEK293T cells. Cells extracts were harvested 24 h after transfection and subjected to co-immunoprecipitation (Co-IP) and immunoblot (IB) analysis. (**B**) PBMCs were treated with 200 ng/ml LPS for 0, 60, and 120 min, and collected at the indicated time points. Cell extracts were harvested for Co-IP with anti-p65/RelA, followed by IB analysis with anti-LRRC25 antibody. Cell extracts without treatment were used as IgG control (Isotype ctrl). (**C**) Domain structures of p65/RelA and domain deletions. RHD, Rel homology domain; TAD, transactivation domain. (**D**) Flag-p65/RelA full length (FL) and deletion mutants were co-transfected with Myc-LRRC25 into HEK293T cells. Cell extracts were immunoprecipitation with anti-Flag and immunoblotted for Myc-LRRC25. (**E**) The structure of LRRC25 and its truncation mutants. (**F**) HEK293T cells were transfected with HA-p65/RelA and Flag-LRRC25 or its truncation mutants. Cells extracts were immunoprecipitation with anti-Flag and immunoblotted for HA-p65/RelA. Repeated experiment was shown in Supplementary Fig. S4. (**G**) Immunoblot analysis of protein extracts of 293T cells transfected with empty vector or vector for Flag-LRRC25 or truncation mutants, along with expression plasmids of HA-p65/RelA. (**H**) HEK293T cells were transfected with a NF-κB-luc reporter plasmid, together with Flag-p65/RelA, an empty vector or LRRC25 (FL) or its truncation mutants. Cells were harvested 24 h after transfection and were subjected to luciferase activity analysis. Data are means ± SEM of three independent experiments (*p < 0.05, **p < 0.01, ***p < 0.001). Experiments for figure **A**–**G** were repeated three times and showed consistent results and repeated blots are shown in Supplementary Figs S2 and S4. Unprocessed original scans of blots are shown in Supplementary Fig. S4.

Since LRRC25 promotes the degradation of p65/RelA, we next investigated how LRRC25 mediates p65/RelA degradation. Coimmunoprecipitation and IB experiments demonstrated that Myc-tagged-LRRC25 interacted with Flag-tagged-p65/RelA in 293T cells (Fig. 5A). To demonstrate the endogenous interaction between LRRC25 and p65/RelA in PBMCs upon LPS stimulation, we treated PBMCs with LPS for different time points, and found the interaction between LRRC25 and p65 in unstimulated cells, but such an interaction was further enhanced after LPS stimulation (Fig. 5B). We next determined the region of p65/RelA responsible for binding to LRRC25 by generating a series of Flag-tagged p65/RelA truncation mutants (Fig. 5C), and found the RHD domain (D1), but not the TAD domain (D2), of p65/RelA interacted with LRRC25 (Fig. 5D).

We also generated several truncation mutants of LRRC25 by deleting the N-terminal LRRs (T1), the LRRs and middle region (T2) or the C-terminal region (T3), and performed immunoprecipitation to determine their interactions with p65/RelA (Fig. 5E). Immunoprecipitation and immunoblot analysis revealed that LRRC25-FL, LRRC25-T1 and LRRC25-T2, but not LRRC25-T3, interacted with p65/RelA (Fig. 5F), suggesting that LRR domain is responsible for its interaction with p65/RelA. Furthermore, we showed that like LRRC25-FL, LRRC25-T1 and T2 also promoted the degradation of p65/RelA, while LRRC25-T3 failed to do so (Fig. 5G). To further determine which LRRC25 domain is required for LRRC25-mediated inhibition, we showed that LRRC25-T1 and -T2, but not LRRC25-T3, strongly inhibited NF-κB-luc activity (Fig. 5H). These results suggest that the LRR domain of LRRC25 is critical for its inhibitory effect on the NF-κB signaling pathway through interacting with p65/RelA.

### LRRC25 promotes the degradation of p65/RelA through autophagy

**Figure 6:**
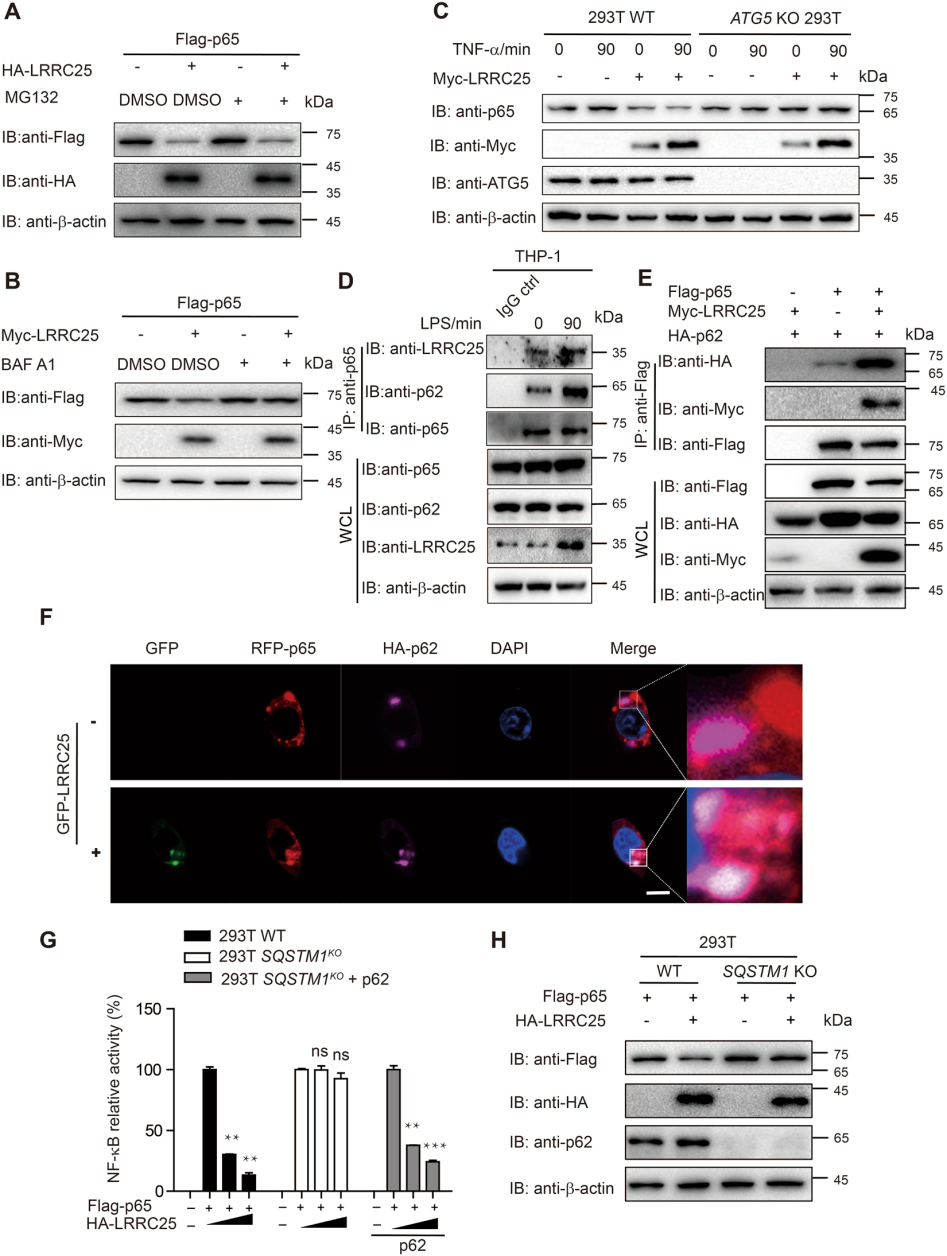
LRRC25 Promotes the Degradation of p65/RelA through Autophagy. (**A**) The expression plasmids of Flag-p65/RelA and HA-LRRC25 were transfected into HEK293T for 24 h. Cells were either untreated or pretreated for 6 h with the proteasome inhibitor MG132 (5 μM), and then cells lysates were subjected to immunoblot analysis. (**B**) HEK293T cells were transfected with the expression plasmids of Flag-p65/RelA and Myc-LRRC25 and either untreated or treated for 6 hrs with Bafilomycin A1 (Baf A1) (100 μM). Cells were harvested 24 h after transfection and analyzed by immunoblot. (**C**) 293T WT cells or *ATG5* KO 293T cells were transfected with Myc-LRRC25, along with TNF-α treatment. Cells were harvested 24 h after transfection and analyzed by immunoblot with the indicated antibodies. **(D)** THP-1 cells were treated with or without LPS and cells extracts were harvested for Co-IP with anti-p65/RelA, followed by IB analysis with anti-LRRC25 or anti-p62 antibodies. Cell extracts without treatment were used as IgG control. (**E**) Flag-p65/RelA, HA-p62 and Myc-LRRC25 expression plasmids were transfected into HEK293T cells. Cells extracts were harvested 24 h after transfection and subjected to immunoprecipitation (IP) and immunoblot (IB) analysis. (**F**) 293T cells were transfected with HA-p62 or GFP-LRRC25 and RFP-p65 for 24 h, and then stained with anti-HA-tag DyLight 650 antibody. DAPI (blue) was used for nuclear staining, Scale bar: 10 μm. (**G**) WT and *SQSTM1* KO cells were transfected with a NF-κB-luc reporter plasmid, together with Flag-p65/RelA, an empty vector or HA-LRRC25 and analyzed for NF-κB luciferase activity. Values are means ± SEM of three independent experiments (*p < 0.05, **p < 0.01, ***p < 0.001). (**H**) HA-LRRC25 or an empty vector and Flag-p65/RelA were co-transfected into 293 T WT cells or *SQSTM1* KO 293T cells. All experiments were repeated for three times and repeated blots are shown in Supplementary Fig. S2. Unprocessed original scans of blots are shown in Supplementary Fig. S5.

It has been reported that p65/RelA undergoes ubiquitination and can be degraded by proteasomal pathway^16^. Therefore, we first assessed whether LRRC25 mediated the degradation of p65/RelA through proteasomal pathway. However, we unexpectedly found that LRRC25 did not degrade p65/RelA through a proteasome pathway, since MG132, an inhibitor of proteasome pathway, failed to block LRRC25-meidated degradation of p65/RelA (Fig. 6A). Therefore, we next investigated if autophagy serves as an alternative mechanism responsible for p65/RelA degradation. Interestingly, LRRC25-mediated degradation of p65/RelA was inhibited in the presence of Bafilomycin A1, a lysosomal inhibitor, which inhibits the fusion between autophagosomes and lysosomes (Fig. 6B). To further determine the role of autophagy in the degradation of p65/RelA by LRRC25, we generated *ATG5* KO 293T cells using the CRISPR/Cas9 system, and found that the degradation of endogenous p65/RelA induced by LRRC25 was completely blocked in *ATG5* KO 293T cells (Fig. 6C). These results suggest that LRRC25 mediates p65/RelA degradation through the autophagy pathway.

It has been reported that p62 (encoded by *SQSTM1*) functions as a major cargo receptor to deliver proteins for degradation to autophagosomes during autophagy^17^. We next determined whether LRRC25 could promote the autophagy degradation of p65/RelA through p62. We showed that the endogenous interactions among p65, LRRC25 and p62 could be enhanced after LPS treatment (Fig. 6D). We next checked whether LRRC25 had any effect on the association between p65/RelA and p62, and found that LRRC25 promoted the interaction between p65/RelA and p62 (Fig. 6E). Moreover, confocal microscopic analysis revealed that ectopic expression of LRRC25 enhanced p65/RelA-p62 co-localization (Fig. 6F). Our data also indicated that the LRRC25 punctate foci formed after stimulation were associated with autophagosomes, since we had observed that LRRC25 was co-localized with LC3 (Supplementary Fig. S1G).

Next, we performed luciferase assay and observed that the inhibitory effect of LRRC25 on NF-κB activation was also abrogated in *SQSTM1* KO cells, but could be restored when p62/SQSTM1 was re-introduced into *SQSTM1* KO cells (Fig. 6G). Consistently, we observed that LRRC25 failed to induce p65/RelA degradation in *SQSTM1* KO 293 T cells (Fig. 6H). Taken together, these data suggest that LRRC25 functions as a bridge to mediate p65/RelA degradation through p62.

## Discussion

The function and regulation of NF-κB has been extensively studied since it was discovered in 1986. NF-κB plays a critical and evolutionarily conserved role in regulating the immune system, leading rapid responses to pathogens, cell differentiation and survival^10^. Activation of the NF-κB signaling pathway results in the upregulated expression of a variety of genes, which are responsible for subsequent inflammatory and immune responses. Increasing evidence suggests that dysregulation of the NF-κB signaling pathway may cause severe chronic inflammation, autoimmunity and cancers^5^. Sustained production of pro-inflammatory cytokines, such as TNF-α, IL-6 and IL-1β, results in chronic inflammation, which, in turn, increases cancer risk, tumor development and progression^18,19^. Therefore, tight regulation of NF-κB signaling pathway is critical and required for the maintenance of homeostasis.

Despite significant progresses, most studies report the negative regulation of NF-κB signaling pathway through inhibiting upstream signaling and IκB proteins. The negative regulation of the NF-κB activation at the p65/RelA has yet to be fully understood. The activation of NF-κB can be suppressed at the p65/RelA level by the following mechanisms: (1) the posttranscriptional modification such as acetylation^20^; (2) replacement of p50/p65 with p52/RelB as an alternative pathway^21^; and (3) degradation of p65/RelA for the inhibition of NF-κB activity^16,22–24^. It has been reported that peroxisome proliferator activated receptor-γ (PPARγ) as well as PDLIM2 (also known as SLIM or mystique) act as E3 ligases to mediate p65/RelA ubiquitination as well as proteasome-dependent degradation^25,26^. Furthermore, another study has demonstrated that the TLR2-dependent signaling from hepatoma-conditioning medium mediates p65/RelA lysosomal degradation^23^.

The LRR domain is commonly found in proteins involved in innate immunity^27,28^. There are at least 375 members of the LRR-containing proteins^14^. However, the functions of most of these LRR-containing proteins currently remain unclear in innate immune responses. In this study, we have identified LRRC25 as a novel negative regulator in the NF-κB signaling pathway, and provided molecular insight into the mechanisms of the innate immune homeostasis maintenance. Our data show that LRRC25 could be significantly upregulated upon activation of the NF-κB signaling pathway stimulated by LPS or TNF-α. Ectopic expression of LRRC25 suppresses NF-κB signaling pathway activated by LPS and TNF-α in a dose-dependent manner. *LRRC25* deficiency in THP-1 cells significantly enhances the activation of NF-κB as well as the inflammatory cytokine production. We further show that the LRR domain of LRRC25 is responsible for its inhibitory effect on NF-κB signaling pathway. In particularly, the LRR domain of LRRC25 interacts with the RHD domain of p65/RelA, and mediates the p65/RelA degradation.

**Figure 7:**
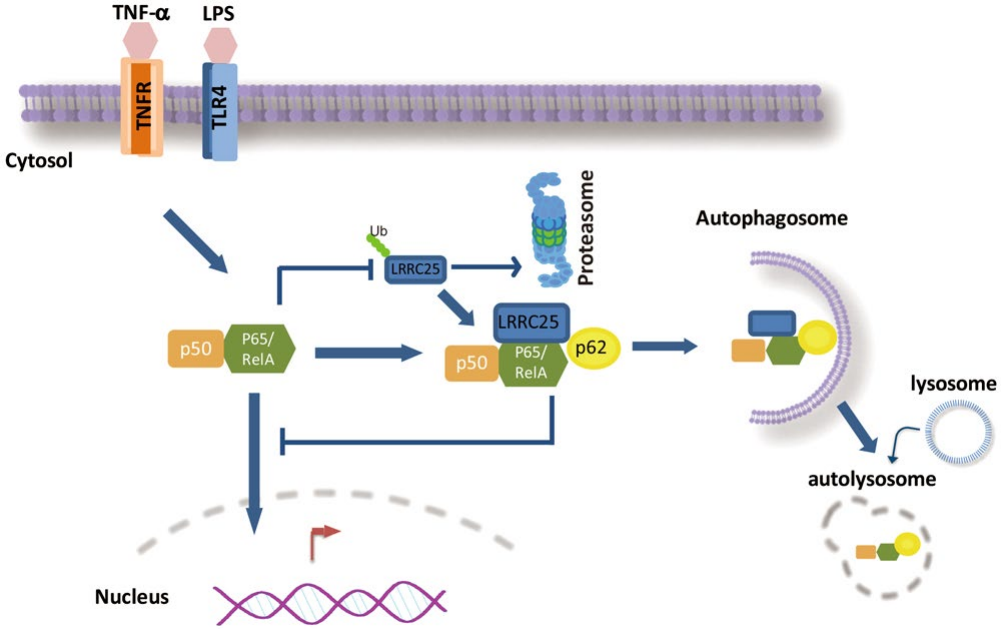
A proposed working model of LRRC25-mediated negative regulation of NF-κB signaling pathway. NF-κB signaling activation stabilizes LRRC25, which, in turn, acts as a bridge to promote the interaction between p65/p50 and p62/SQSTM1 for feedback negative regulation of p65/Rel, leading to the degradation of p65/RelA through autophagic pathway and inhibiting NF-κB signaling.

Autophagy is a highly conserved process in eukaryotic cells that enables intracellular organelles and misfolded proteins to be digested in lysosomes^29^. Autophagy is important to maintain the cellular hemostasis and has multiple important effects on immunity^17,30,31^. Upon various environmental stress, such as starvation, radiation and pathogen infection, autophagy initiates with the formation of double-membrane vesicles called autophagosomes, leading to the degradation of cytosolic components by acid hydrolase in the lysosomes^17,31^. Our studies show that LRRC25-mediated p65 degradation is dependent on autophagy pathway. ATG5 deficiency abolishes LRRC25-mediated p65/RelA degradation. To understand how LRRC25 promotes autopahagic degradation of p65/RelA, we demonstrate that LRRC25 may serve as a bridge to enhance the association between p65/RelA and p62/SQSTM1. p62/SQSTM1 is a well-known cargo receptor in autophagy by recognizing the ubiquitinated proteins for selective degradation via its ubiquitin-binding domain^17,32^. Based on our results, we proposed a working model to explain how LRRC25 exerts its inhibitory effect on NF-κB signaling pathway by targeting p65/p50 (Fig. 7). NF-κB signaling activation will stabilize LRRC25 protein, which, in turn, mediates a feedback negative regulation of p65/RelA by promoting the interaction between p65/p50 and p62 for autophagic degradation. Overall, our findings have identified a previously unrecognized role of LRRC25 in innate immune signaling and have provided a novel mechanism of the negative regulation of p65/RelA in NF-κB signaling pathway. Thus, this study underlines the potential of LRRC25 as a potential target for new therapeutic treatment against infectious and inflammation-associated diseases.

## Materials and Methods

### Cell culture and transfection

THP-1 cells, HeLa cells, and Human embryonic kidney 293T (HEK293T) cells were purchased from American Type Culture Collection. Peripheral blood mononuclear cells (PBMCs), THP-1 cells, HeLa cells, and HEK293T were maintained in DMEM (CORNING) or RPMI-1640 medium (CORNING) containing 10% fetal bovine serum (Gibco) as described (Cui *et al*. 2016). Plasmids were transfected with Lipofectamine 2000 reagent (Invitrogen) according to the manufacturer’s instructions.

### Antibodies and reagents

The antibodies used in this study were as follows: HRP-anti-hemagglutinin (12013819001) and anti-c-Myc-HRP (11814150001) (Roche Applied Science); HRP-anti-Flag (M2) (A8592) and anti-β-actin (A1978) (Sigma-Aldrich, St. Louis, MO); anti-phospho-IKKα/β (No. 2697s), anti-phospho-JNK (No.9251), anti-JNK (No. 9252) anti-phospho-ERK (No. 9101), anti-ERK (No. 9102), anti-phospho-ERK (No. 9101), anti-ERK (No. 9102), anti-phospho-p38 (No. 9211), anti-p38 (No. 9212), anti-p65/RelA (No. 6956), anti-IκBα (No. 4814) and anti-phospho-IkBα (Ser32/36) (No. 9246) (Cell Signaling Technology, Danvers, MA)); anti-IKK (Merck Millipore, Billerica, MA); Anti-LRRC25 (ab84954) (Abcam, Cambridge, U.K.); Mouse mAb HA tag DyLight 650(ab117515) (Abcam, Cambridge, U.K.). Recombinant human TNF-α was purchased from PeproTech; Lipopolysaccharides (LPS) (L4391-1 MG) and Bafilomycin A1 were purchased from Sigma-Aldrich (St. Louis, MO). Trizol was purchased from Invitrogen and Transcript First Strand cDNA Synthesis Kit from Roche.

### Luciferase reporter assays

HEK293T (2 × 10^5^) cells were plated in 96-well plates and transfected with plasmids encoding a NF-κB luciferase reporter (firefly luciferase plasmid; 5 ng), and *pRL-TK-luc* (renilla luciferase plasmid; 10 ng) together with 5 ng plasmids encoding Flag-MyD88, Flag-TRAF6, Flag-TAK1 + HA-TAB1, Flag-IKKα, Flag-IKKβ and Flag-p65/RelA, along with increasing concentrations (0, 50, or 100 ng) of plasmids expressing LRRC25 or the domain deletions of LRRC25. Cells were harvested at 24 h after transfection and luciferase activity was measured with the Dual-Luciferase Assay kit according to the manufacturer’s protocol (Promega). The reporter gene activity was determined based on normalization of firefly luciferase activity to renilla luciferase activity.

### Quantitative real-time PCR

Total RNA was isolated using TRIzol reagent (Life Technologies, Gaithersburg, MD) and reverse transcribed using oligo-dT primers and reverse transcriptase (TAKARA). Real-time quantitative PCR was performed with SYBR green qPCR Mix kit (Genstar) and specific primers using the Primer 5.0 analyzer (Applied Biosystems). *GAPDH* was used as a reference gene. The following primers were used for qPCR analysis:

*GAPDH*: Forward, 5′-ACAACTTTGGTATCGTGGAAGG-3′,

Reverse, 5′-GCCATCACGCCACAGTTTC-3′;

*IL-1*β: Forward, 5′-ATGATGGCTTATTACAGTGGCAA-3′

Reverse, 5′-GTCGGAGATTCGTAGCTGGA-3′;

*IL-6*: Forward, 5′-AGAGGCACTGGCAGAAAACAAC-3′,

Reverse, 5′-AGGCAAGTCTCCTCATTGAATCC-3′;

*TNF-*α: Forward, 5′-CCAGACCAAGGTCAACCTCC-3′,

Reverse, 5′-CAGACTCGGCAAAGTCGAGA-3′;

*CCL20*: Forward, 5′-TGATGTCAGTGCTGCTACTC-3′,

Reverse, 5′-ATGTCACAGCCTTCATTGGC-3′;

*MnSOD*: Forward, 5′-AACGCGCAGATCATGCA-3′,

Reverse, 5′-CTCCCAGTTGATTACATTC-3′;

*ICAM-1*: Forward, 5′-AGACCTTAGCGCGGTGTAGA-3′,

Reverse, 5′-AGTAGCAGAGGAGCTCAGCG-3′.

### Immunoprecipitation and immunoblot analysis

Proteins were gently extracted in ice-cold low-salt lysis buffer as described (Yang *et al*.^8^). For immunoprecipitation, whole cell extracts were prepared after transfection or stimulation with appropriate ligands, followed by incubation overnight with the appropriate antibodies plus anti-Flag, anti-hemagglutinin agarose gels (Sigma), or Protein A/G beads (Pierce). Beads were washed six times with low-salt lysis buffer (50 mM Hepes pH 7.5, 150 mM NaCl, 1 mM EDTA, 1.5 mM MgCl2, 10% glycerol, 1% Triton X-100) supplemented with 5 mg/ml protease inhibitor cocktail (Roche) and immunoprecipitates were re-suspended with 3× SDS Loading Buffer (FD Biotechnology).The released proteins were equally loaded on 8–12% SDS-polyacrylamide gel and transferred onto PVDF membranes (Bio-Rad). Membranes were incubated with specific antibodies, and detected using enhanced chemiluminescence (Millipore). Every experiment was repeated at least three times and got consistent results and repeated blots are shown in the supplementary information.

### Generation of knockout cells by the CRISPR/Cas9 technology

*LRRC25*, *ATG5* and *SQSTM1* knockout cells were generated by the CRISPR/Cas9 system, and the sequences of target sgRNA are as follows:

*LRRC25*-guide RNA: 5′-CACCGTGTCCTCCGCGGATGTGG-3′,

*ATG5*-guide RNA: 5′-GTGCTTCGAGATGTGTGGTT-3′,

*SQSTM1*-guide RNA: 5′-TCAGGAGGCGCCCCGCAACA-3′.

### Fluorescence microscopy

Cells were cultured on a glass-bottomed dish and transfected with indicated plasmids for 24 h, and then cells were fixed with 4% paraformaldehyde for 20 min, and then stained with specific antibodies. Localization images were examined under Zeiss LSM 780 (Carl Zeiss, Germany) and acquired using ZEN 2009 light Edition software (Carl Zeiss, Germany).

### Statistical analyses

The results of all quantitative experiments are reported as mean ± SEM of three independent experiments, and a two-tailed Student’s *t*-test was used for all statistical analyses with the GraphPad Prism 5 software (GraphPad Software, La Jolla, CA, US). Differences between two groups were considered significant when *P*-value was < 0.05.

## Electronic supplementary material

Suppementary information
