# Supplementary material for: LRRC25 Functions as an Inhibitor of NF-κB Signaling Pathway by Promoting p65/RelA for Autophagic Degradation
Source: Sci Rep. 2017 Oct 18;7:13448. doi: 10.1038/s41598-017-12573-3 (PMC5647368; doi:10.1038/s41598-017-12573-3)

## Supplementary Information

# LRRC25 Functions as an Inhibitor of NF-κB Signaling Pathway by Promoting p65/RelA for Autophagic Degradation

Yanchun Feng[1,2,6], Tianhao Duan[1,2,6], Yang Du[1,2,6], Shouheng Jin[2], Jun Cui[2*], and Rong-Fu Wang[3-5*]

[1]Zhongshan School of Medicine, Sun Yat-sen University, Guangzhou, 510080, China

[2]Key Laboratory of Gene Engineering of the Ministry of Education, State Key Laboratory of Biocontrol, School of Life Sciences, Sun Yat-sen University, Guangzhou, 510275, China. [3]Center for Inflammation and Epigenetics, The Methodist Hospital Research Institute, Houston, 77030, Texas, USA. [4]Department of Microbiology and Immunology, Weill Cornell Medical College, Cornell University, New York, NY 10065, [5]Institute of Biosciences and Technology, College of Medicine, Texas A & M University, Houston, Texas 77030, USA.

[6]These authors contributed equally to this work.

***Corresponding author:** Jun Cui, School of Life Sciences, Sun Yat-sen University, Guangzhou, Guangdong, P.R. China, Tel: +86 20 39943429; E-mail: cuij5@mail.sysu.edu.cn, and Rong-Fu Wang, Center for Inflammation and Epigenetics, The Methodist Hospital Research Institute, Houston, Texas, USA. Tel: +1 713 441 7359; E-mail: rwang3@houstonmethodist.org

## Supplementary Figure legends

## Supplementary Figure S1

**(A)** Extracts of 293T cells transfected with plasmids for HA-tagged LRRC proteins and analyzed by immunoblotting. **(B)** THP-1 cells were treated with LPS (200 ng/ml) at the indicated time points and the mRNA level of *LRRC25* was detected by real-time PCR analysis. **(C)** Immunoprecipitation and immunoblot analysis of 293T cells transfected with empty vector or vectors for Myc-p65, together with Flag-LRRC25 and HA-K48. **(D)** Immunoblot analysis of protein extracts of 293T cells transfected with empty vector or vectors for HA-LRRC25 in an increasing amount (wedge), along with expression plasmids of Flag-p65/RelA. Below, real-time analysis of *RelA* mRNA: *GAPDH* (encoding glyceraldehyde phosphate dehydrogenase) was used as a loading control. **(E)** THP-$1^{WT}$ and *LRRC25*$^{KO}$ THP-1 cells were treated with LPS (200 ng/ml) at the indicated time points. The expression of p65/RelA and phospho-IκB, total IκB, phospho-IKK, total IKK, phospho-JNK, total JNK, phospho-ERK, total ERK, phospho-p38, total p38 were analyzed by immunoblotting with the indicated antibodies. **(F)** Immunoblot analysis of 293T cells transfected with plasmids for Flag-tagged p65, p105 or p100, together with HA-tagged LRRC25. **(G)** Confocal microscopy of HeLa cells transfected with GFP-LC3 and HA-LRRC25 for 24 hrs with or without 20 ng/ml TNF-α treatment for 90 min. Cells were fixed and stained with anti-HA-tag DyLight 650 antibody. DAPI (blue) was used for nuclear staining, Scale bar: 10 μm.

## Supplementary Figure S2

The repeated blots for figures 1C, 4C, 5A, 5B, 5D, 5G, 6A, 6B, 6C, 6D, 6E, and 6H.

## Supplementary Figure S3

The full blot images, with molecular weight markers indicated, for figures 1C, 2C, 4A, 4C and 4D.

## Supplementary Figure S4

The full blot images, with molecular weight markers indicated, for figures 5A, 5B, 5D, 5F and 5G.

## Supplementary Figure S5

The full blot images, with molecular weight markers indicated, for figures 6A, 6B, 6C, 6D and 6G.

# Supplementary Information

## Supplementary Figure S1

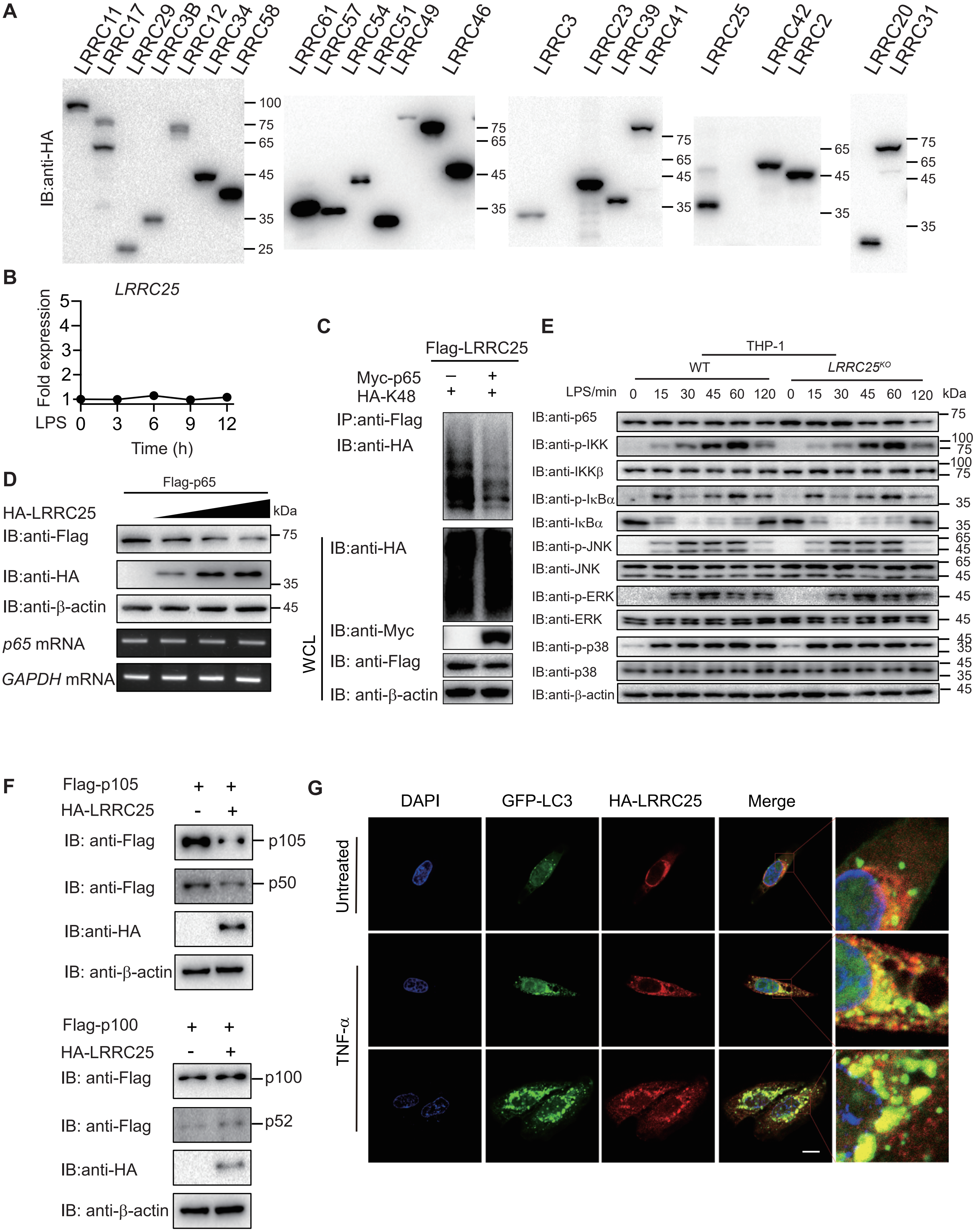

Supplementary Information

Supplemetaty Figure S2 The repeated blots for figures 1C, 4C, 5A, 5B, 5D, 5G, 6A, 6B, 6C, 6D, 6E, and 6H.

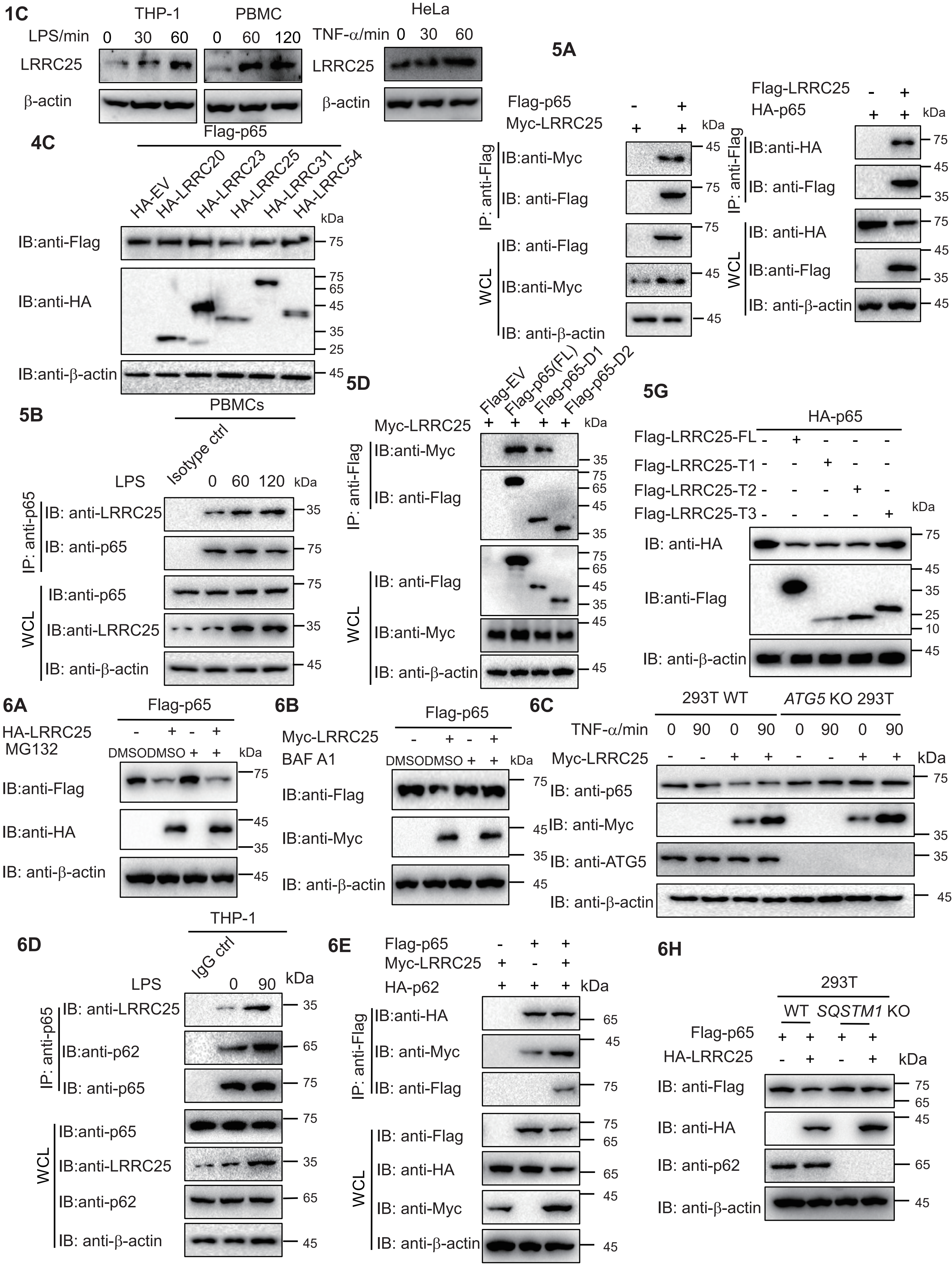

# Supplementary Information

Supplementary Figure S3. The full blot images, with molecular weight markers indicated, for Figures 1C, 2C, 4A, 4C and 4D

Fig.1c

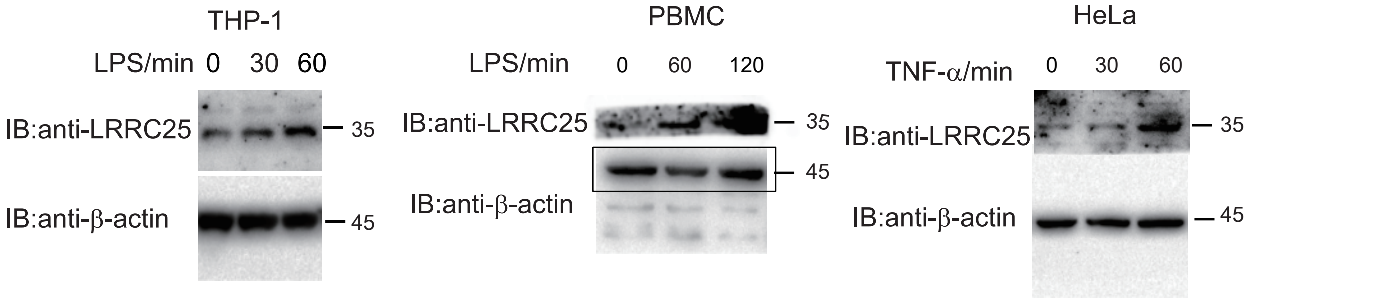


Fig.2c

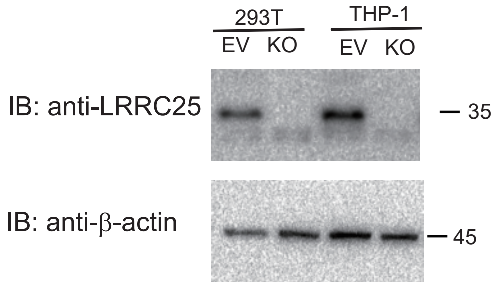


Fig.4d

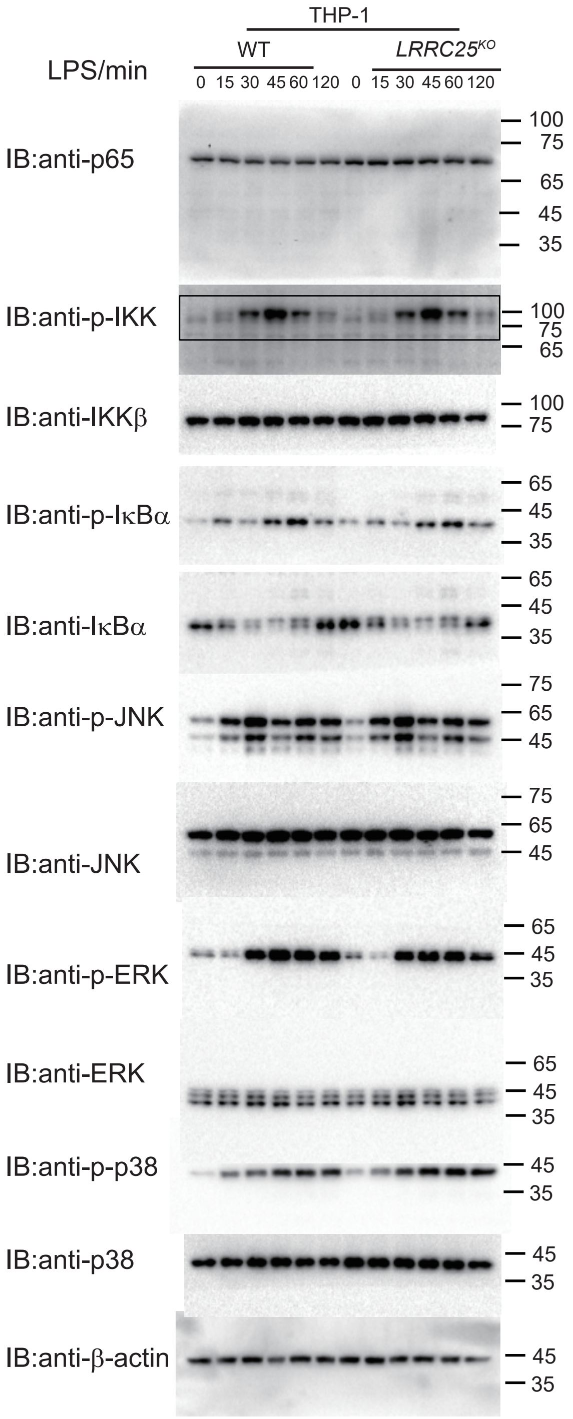


Fig.4a

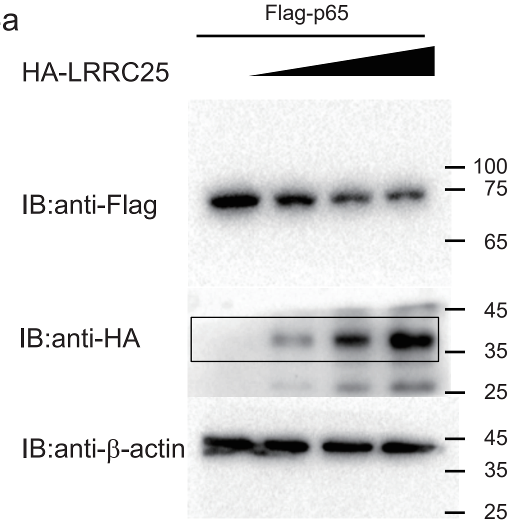


Fig.4c

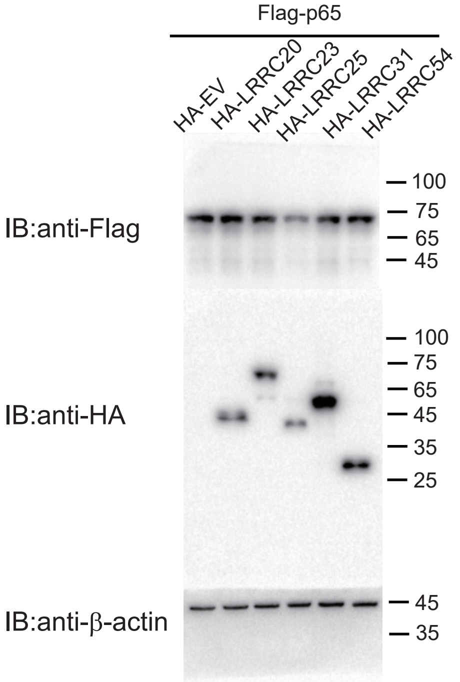

Supplementary Information

Supplementary Figure S4. The full blot images, with molecular weight markers indicated, for Figures 5A, 5B, 5D , 5F and 5G

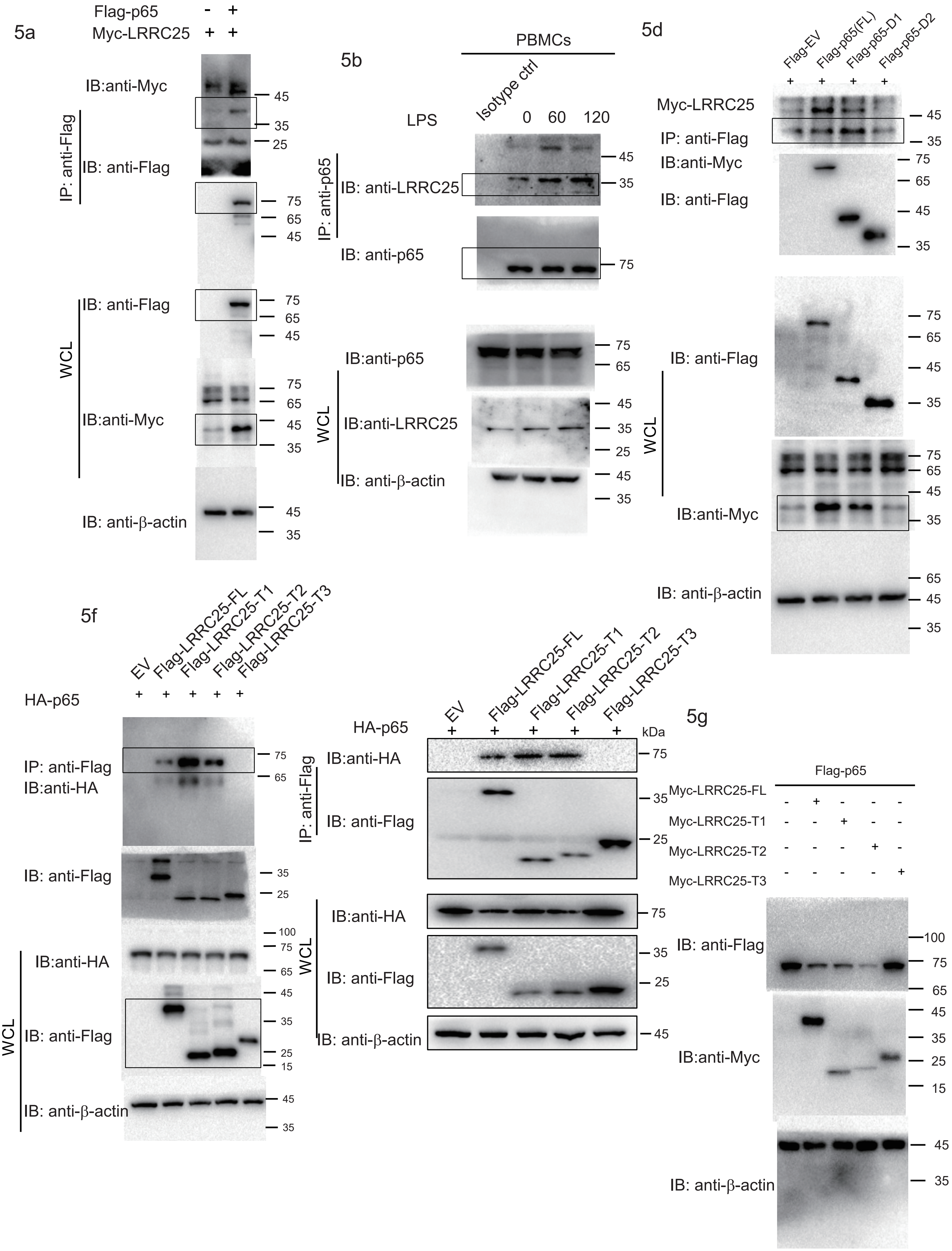

Supplementary Information

Supplementary Figure S5. The full blot images, with molecular weight markers indicated, for Figures 6A, 6B, 6C , 6D and 6G.

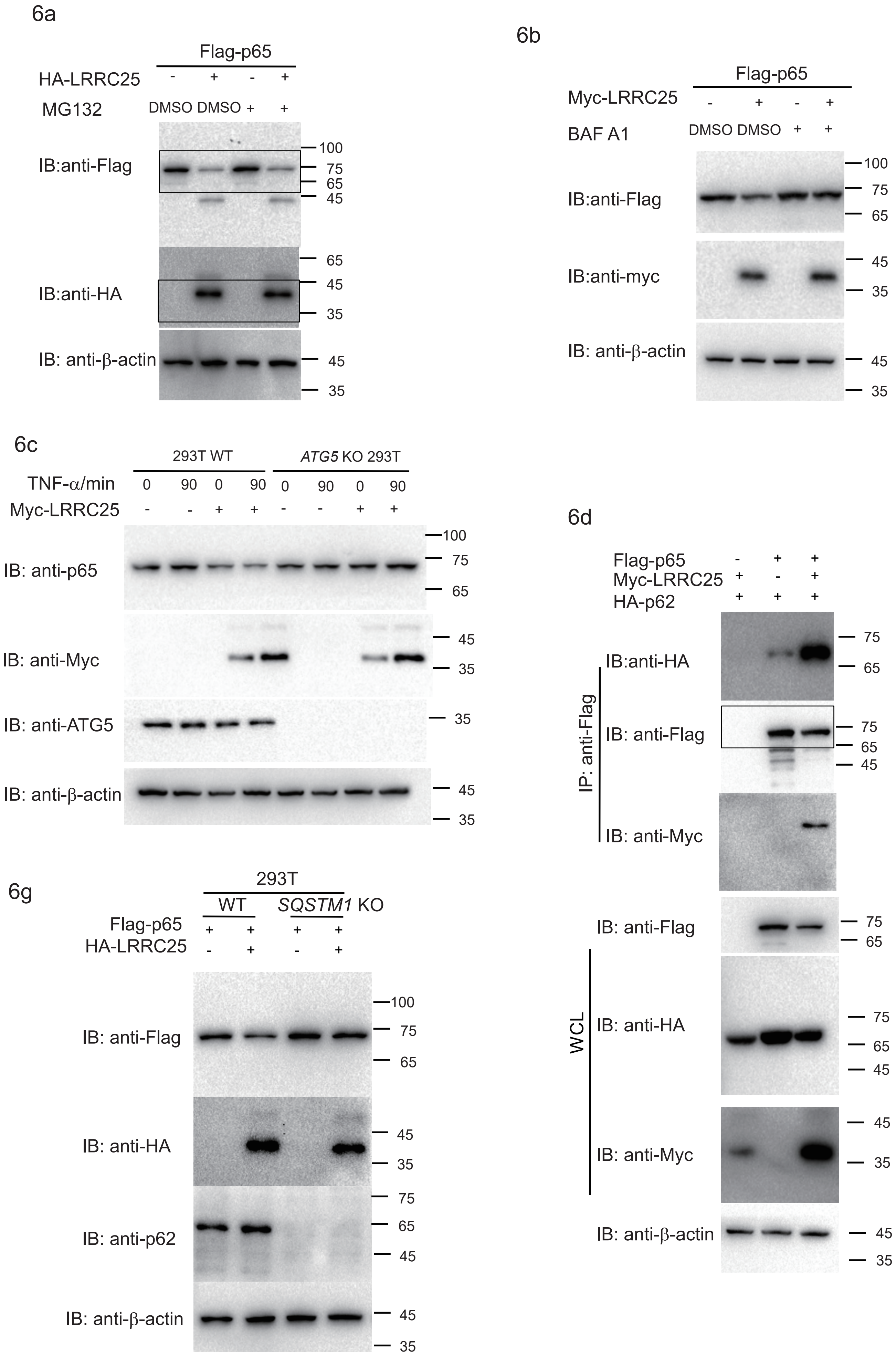

Supplement: Supplementary file 1 — Suppementary information [file 41598_2017_12573_MOESM1_ESM.pdf]
